# Supplementary material for: Silver Nanoparticle-Embedded Conductive Hydrogels for Electrochemical Sensing of Hydroquinone
Source: Polymers (Basel). 2023 May 23;15(11):2424. doi: 10.3390/polym15112424 (PMC10255235; doi:10.3390/polym15112424)
Supplement: Supplementary file 1 [file polymers-15-02424-s001.zip › polymers-2328326-supplementary.pdf]

Support information

Silver nanoparticles-embedded conductive hydrogels for electrochemical sensing of hydroquinone

Tingting Xu[12,*], Huanli Gao[1], Orlando J.Rojas[2], Hongqi Dai[1,*]

[1] *Jiangsu Co-Innovation Center of Efficient Processing and Utilization of Forest Resources, International Innovation Center for Forest Chemicals and Materials, College of Light Industry and Food Engineering, Nanjing Forestry University, Nanjing 210037, China*

[2] *Bioproducts Institute, Departments of Chemical and Biological Engineering, Chemistry and Wood Science, The University of British Columbia, 2360 East Mall, Vancouver, BC V6T 1Z3, Canada*

* Corresponding Authors.
E-mail: salyting@njfu.edu.cn (T.X.); hgdhq@njfu.edu.cn (H.D.)

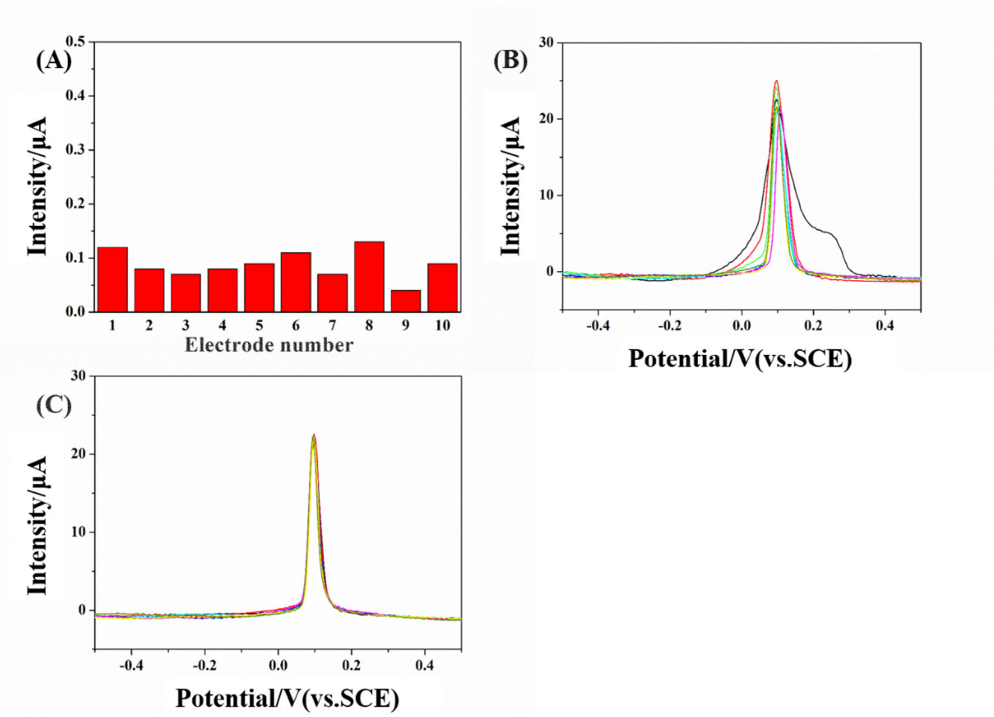

Figure S1. (A) Current intensity response of Ag NP-CH/GCE in blank solution for ten consecutive times, (B) DPVs of Ag NP-CH/GCE for eight different electrodes with 30 μM HQ and (C) DPVs of obtained on one electrode which was kept in Tris-HCl buffer solution at 4°C for one week and detected every other day with 30 μM HQ.
